# *Baylisascaris procyonis*: An Emerging Helminthic Zoonosis

**DOI:** 10.3201/eid0804.010273

**Published:** 2002-04

**Authors:** Frank Sorvillo, Lawrence R. Ash, O.G.W. Berlin, JoAnne Yatabe, Chris Degiorgio, Stephen A. Morse

**Affiliations:** * University of California Los Angeles School of Public Health, Los Angeles, California; † Specialty Labs, Santa Monica, California, USA; ‡ University of California Los Angeles School of Medicine, Los Angeles, California, USA; § Centers for Disease Control and Prevention, Atlanta Georgia, USA

**Keywords:** *Baylisascaris procyonis*, larva migrans, epidemiology

## Abstract

*Baylisascaris procyonis*, a roundworm infection of raccoons, is emerging as an important helminthic zoonosis, principally affecting young children. Raccoons have increasingly become peridomestic animals living in close proximity to human residences. When *B. procyonis* eggs are ingested by a host other than a raccoon, migration of larvae through tissue, termed larval migrans, ensues. This larval infection can invade the brain and eye, causing severe disease and death. The prevalence of *B*. *procyonis* infection in raccoons is often high, and infected animals can shed enormous numbers of eggs in their feces. These eggs can survive in the environment for extended periods of time, and the infectious dose of *B. procyonis* is relatively low. Therefore, the risk for human exposure and infection may be greater than is currently recognized.

*Baylisascaris procyonis*, a ubiquitous roundworm infection of raccoons (*Procyon lotor*), is increasingly being recognized as a cause of severe human disease (*1*,*2*). *B. procyonis* has a widespread geographic distribution, with infection rates as high as 70% in adult raccoons and exceeding 90% in juvenile raccoons (*3*). As with other ascarids, eggs are excreted in feces and must develop externally, typically in soil, to become infectious. When raccoons ingest infective eggs, larvae will hatch, enter the wall of the small intestine, and subsequently develop to adult worms in the small bowel. However, ingestion of eggs by other host animals, especially rodents and other small mammals, results in extraintestinal migration of larvae (*4*); an estimated 5%-7% of larvae invade the brain (*5*). The migration of helminth larvae through tissue in suboptimal hosts is termed larva migrans and may affect the viscera (visceral larva migrans [VLM]), the eye (ocular larva migrans [OLM]), or the nervous system (neural larva migrans [NLM] (*6*). Raccoons may also become infected when they eat larvae that have become encapsulated in the tissues of rodents and other animals (*3*).

More than 90 species of wild and domesticated animals have been identified as infected with *B. procyonis* larvae (*3*). Outbreaks of fatal central nervous system disease caused by *B. procyonis* have occurred on farms and in zoos and research animal colonies and have affected commercial chickens, bobwhite quail, guinea pigs, commercial pheasants, and domestic rabbits (*7*–*11*). Natural infections have also been recognized in dogs, rodents, porcupines, chinchillas, prairie dogs, primates, woodchucks, emus, foxes, and weasels (*12*–*16*). Experimental infection of a variety of nonhuman primates has also been reported (*17*).

## Human Infection

*B. procyonis* infection of humans typically results in fatal disease or severe sequelae (*1**,**2**,**18**-**24*; pers. comm., W. Murray). Clinical manifestations include eosinophilic encephalitis, ocular disease, and esoinophilic cardiac pseudotumor. Elevated peripheral cerebrospinal fluid eosinophilia can be detected in cases of meningoencephalitis. Eleven recognized human cases, four of them fatal, have been reported (Table). The first human case was reported in 1984 in a 10-month-old infant with fatal eosinophilic meningoencephalitis (*18*). At autopsy, numerous granulomas containing larvae of *B. procyonis* were observed in several organs and tissues (*18*). The brain was the most heavily affected, with granulomas concentrated in the periventricular white matter, around the dentate nuclei, and along the cerebral and cerebellar cortices. Numerous granulomas and larvae were also found in the mesentery and cardiac tissue. The infant’s family lived in a rural, wooded area of Pennsylvania, and raccoons were nesting in unused chimneys at the time infection was acquired.

**Table:** Reported human cases of larval *Baylisascaris procyonis* infection

Year^a^	Location	Age	Sex	Clinical	Outcome	Reference
1980	Pennsylvania	10 mo	Male	Eosinophilic meningoencephalitis	Fatal	*17*
1984	Illinois	18 mo	Male	Eosinophilic meningoencephalitis	Fatal	*18*
1990	New York	13 mo	Male	Eosinophilic meningoencephalitis	Severe neurologic sequelae	*19*
1992	California	29 yr	Male	Diffuse unilateral subacute neuroretinitis	Ocular sequelae	*21*
1991	Germany	48 yr	Female	Diffuse unilateral subacute neuroretinitis	Ocular sequelae	*22*
1995	Massachusetts	10 yr	Male	Esoinophilic cardiac pseudotumor	Fatal	*20*
1996	Michigan	6 yr	Male	Chorioretinitis, neurologic deficits	Severe neurologic sequelae	*23*
1996	Michigan	2 yr	Male	Eosinophilic meningoencephalitis, chorioretinitis	Severe neurologic sequelae	*23*
1997	California	13 mo	Male	Eosinophilic meningoencephalitis	Severe neurologic sequelae	*2*
1998	California	11 mo	Male	Eosinophilic encephalitis	Severe neurologic sequelae	*1*
1999	California	17 yr	Male	Eosinophilic meningoencephalitis	Fatal	^b^

^a^Year of onset or report. ^b^ Pers. comm., W. Murray.

**Figure 1:**
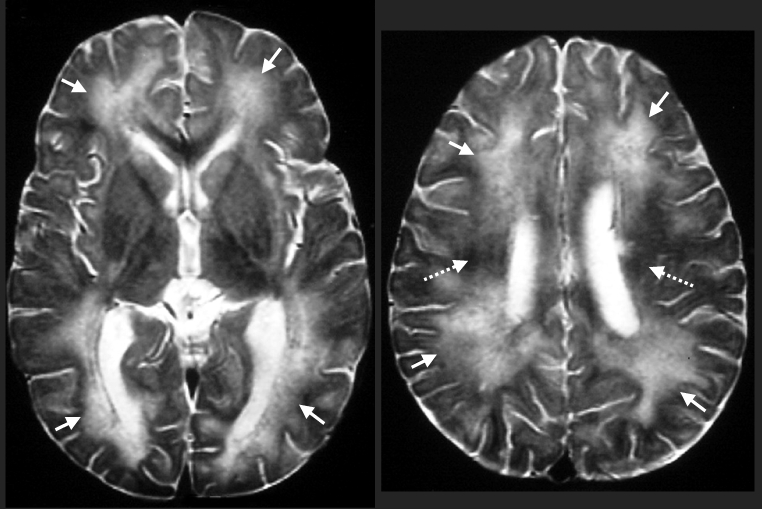
Biopsy-proven *Baylisascaris procyonis* encephalitis in a 13-month-old boy. Axial T2-weighted magnetic resonance images obtained 12 days after symptom onset show abnormal high signal throughout most of the central white matter (arrows) compared with the dark signal expected at this age (broken arrows).

Four additional cases of eosinophilic encephalitis with similar pathologic characteristics have been documented. Magnetic resonance images from a human case of *Baylisascaris* encephalitis are shown in Figure 1. In patients who have survived central nervous system (CNS) invasion, severe neurologic sequelae have resulted. In a fatal case, an eosinophilic cardiac pseudotumor, affecting principally the left ventricle, was observed at autopsy; no larvae or granulomas were found in any other tissue examined.

No effective therapy exists for the visceral form of *B. procyonis* larval infection. In an experimental model, mice treated with albendazole and diethylcarbamezine within 10 days after infection were protected from CNS disease (*25*); however, several anthelminthic agents have been used to treat human cases without success. Laser photocoagulation has been successful in treating ocular infection (*26*).

Because the disease is transmitted by the fecal-oral route, human cases of *B. procyonis* infection typically occur in younger age groups, mainly infants, who often engage in oral exploration of their environment and are therefore more likely to be exposed to *B. procyonis* eggs. Raccoon activity near the patient’s residence is often described. All but one of the reported patients to date have been male; however; there is no reason to believe that females are less susceptible to infection.

## Diagnosis and Underrecognition of Infection

**Figure 2:**
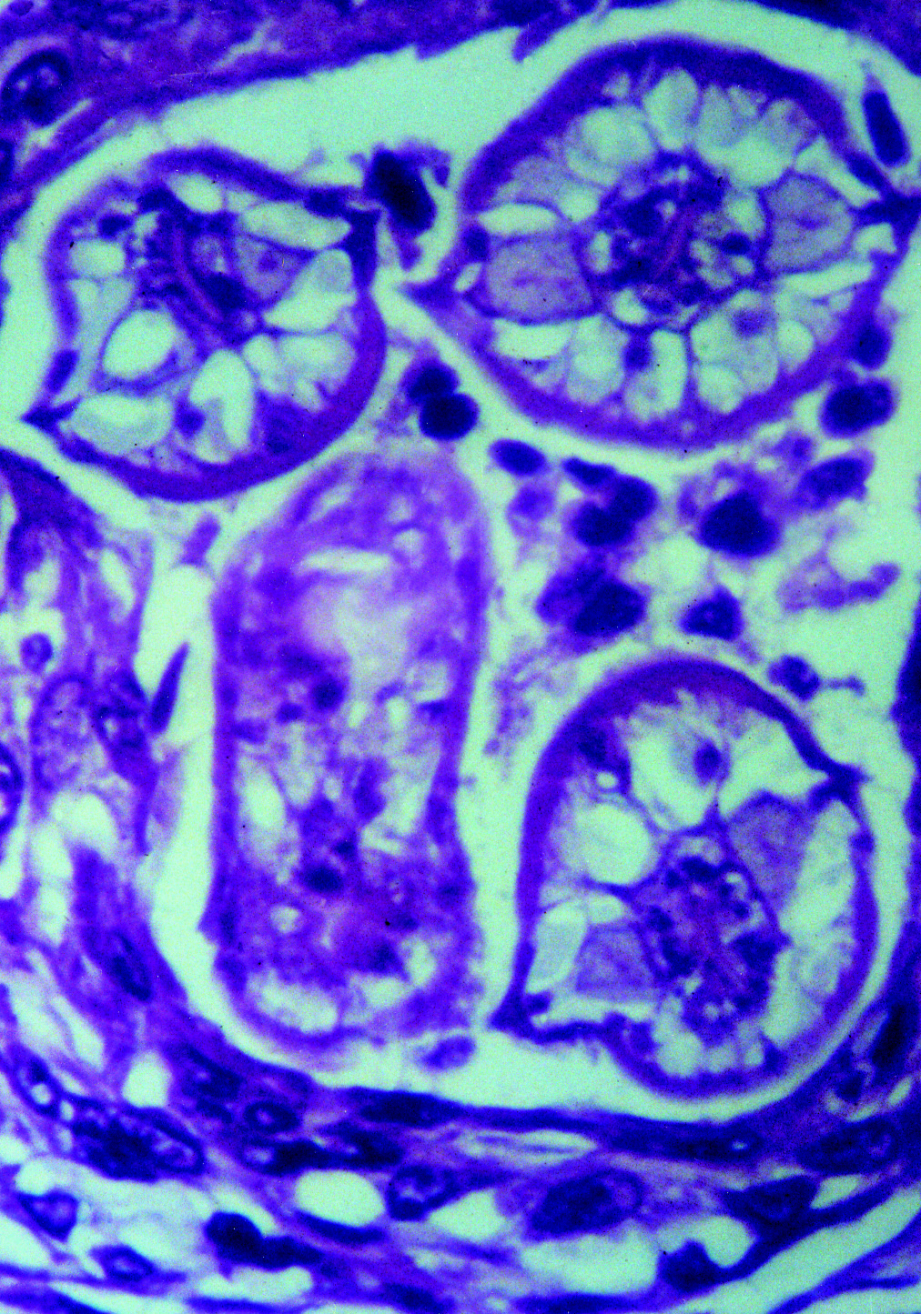
Cross-section of *Baylisascaris procyonis* larva in tissue section of brain, demonstrating characteristic diagnostic features including prominent lateral alae and excretory columns.

Diagnosis of *B. procyonis* infection is typically done through morphologic identification of larvae in tissue sections (*27*). However, accurate diagnosis requires experience in recognizing larval morphologic characteristics and differentiating among a number of possible larval nematode agents, including *Toxocara canis*, *T. cati*, *Ascaris lumbricoides*, and species of *Gnathastoma*, *Angiostrongylus*, and *Ancylostoma,* as well as larval cestode infections such as cysticercosis and echinococcosis (*6*,*27*). Characteristic features of *B. procyonis* larvae in tissue include its relatively large size (60 μ) and prominent single lateral alae (*27*) (Figure 2). While serologic testing has been performed in some cases as supportive diagnostic evidence, no commercial serologic test is currently available (*28*,*29*). However, a presumptive diagnosis can be made on the basis of clinical (meningoencephalitis, diffuse unilateral subacute neuroretinitis [DUSN], pseudotumor), epidemiologic (raccoon exposure), radiologic (white matter disease), and laboratory results (blood and CNS eosinophilia).

Human baylisascariasis is probably underrecognized, and the full spectrum of clinical illness is unclear. The agent is unknown to most clinicians and typically is not considered in a differential diagnosis. In addition, confirming the diagnosis requires an effective biopsy specimen that must contain an adequate cross-section of a larva. Since small numbers of larvae can cause severe disease and larvae occur sporadically in tissue, a biopsy may frequently fail to include larvae; such a specimen will result in a negative finding. Moreover, larval morphologic characteristics may not be recognized or may be misidentified. The accurate diagnosis of parasites in tissues can be difficult even for trained microscopists, and mistaken identification, particularly of helminth larvae, is not uncommon (*27*). Finally, no commercial serologic test exists for the diagnosis of *B. procyonis* infection, and the sensitivity, specificity, and predictive value of available serologic tests are unknown. Evidence for underrecognition of larval *B. procyonis* infection can be found in several reported cases of DUSN caused by larvae compatible with *B. procyonis* and a case of eosinophilic meningoencephalitis reported in an infant in 1975 (*26*,*30*,*31*).

## Infection Potential and Human Risk

Although relatively few human cases of baylisascariasis have been reported, several factors suggest that the likelihood of exposure and infection may be greater than is currently recognized. Raccoons have a widespread geographic distribution, and infection with *B. procyonis* is common in raccoon populations, with typically high prevalence rates observed. An infected raccoon can harbor numerous adult worms and may excrete large numbers of eggs. A single adult female worm may produce an estimated 115,000 to 877,000 eggs per day, and an infected raccoon can shed as many as 45,000,000 eggs daily (*3**,**4**,**32*). In light of the relatively low infectious dose of *B. procyonis* (estimated to be <5,000 eggs) and the viability of the eggs in the environment for months to years, the infection potential is not insubstantial. Raccoons have increasingly become peridomestic animals living in close proximity to human residences and are among the fastest growing wildlife populations nationwide. These animals benefit from feeding on abundant pet food left accessible, either accidentally or intentionally, and their populations can thrive under such conditions. In one suburban area near the residence of a recent patient in northern California, the raccoon population was measured at 30 animals per quarter acre. Areas frequented by raccoons and used for defecation were found in close proximity to human dwellings, and *B. procyonis* eggs are routinely recovered from these areas (*1*). Children, particularly toddlers, may be at particular risk of exposure.

Although baylisascariasis may indeed be underdiagnosed, asymptomatic human infection may be the typical response, and the limited number of cases reported may indicate that an unrecognized immune defect is necessary for severe infection to occur. The prevalence of asymptomatic infection in human populations has yet to be determined.

## A Possible Agent of Bioterrorism

In an era of increasing concern about bioterrorism (*33*), certain characteristics of *B. procyonis* make it a feasible bioterrorist agent. The organism is ubiquitous in raccoon populations and therefore easy to acquire. Enormous numbers of eggs can be readily obtained, and these eggs can survive in an infectious form for prolonged periods of time. As with other ascarids, the eggs can remain viable in a dilute (0.5%-2%) formalin solution for an indefinite period of time, and animal studies suggest that *B. procyonis* has a relatively small infectious dose. Moreover, the organism causes a severe, frequently fatal infection in humans, and no effective therapy or vaccine exists. Introduction of sufficient quantities of *B. procyonis* eggs into a water system or selected food products could potentially result in outbreaks of the infection. A similar agent, *Ascaris suum*, a roundworm of pigs, was used to intentionally infect four university students who required hospitalization after eating a meal that had been deliberately contaminated with a massive dose of eggs (*34*). Contamination of community water sources would be difficult since the eggs of *B. procyonis* are relatively large (80 μm long by 65 μm wide) and would be readily removed by standard filtration methods or the flocculation and sedimentation techniques used by municipal water systems in the United States. However, posttreatment contamination or targeting of smaller systems could be possible.

## Conclusion

Baylisascariasis is an emerging helminthic zoonosis with the potential for severe infection that may be a more important public health problem than is currently recognized. Educating the medical community is of paramount importance in helping to define the extent of infection. Physicians should consider *B. procyonis* infection in the differential diagnosis of patients with eosinophilic meningoencephalitis, DUSN, and eosinophilic pseudotumor. While infants and children have a higher probability of infection, all age groups are at risk. The public should be made aware of the potential risks of exposure to raccoons and raccoon feces. Raccoons should be discouraged as pets or should be routinely evaluated for *B. procyonis* infection and treated. However, screening and treatment may not be sufficient to prevent exposure, since the likelihood of reinfection is high. The public should be discouraged from feeding raccoons and should ensure that possible food sources (such as pet food, water, and garbage) are protected from raccoon access. Further study of the impact of larval *B. procyonis* infection on human health is warranted. Development of a standardized serologic test for *B. procyonis* would allow epidemiologic studies of its prevalence and incidence and help determine factors associated with infection. A sensitive and specific test would also provide a noninvasive method of diagnosis. Finally, a better understanding of the pathogenesis of *B. procyonis* infection and efforts to develop effective treatment approaches are warranted.
